# μ-Carbonato-bis­(bis­{2-[(diethyl­amino)­meth­yl]phen­yl}bis­muth(III))

**DOI:** 10.1107/S160053681005453X

**Published:** 2011-01-12

**Authors:** Albert P. Soran, Mihai G. Nema, Hans J. Breunig, Cristian Silvestru

**Affiliations:** a Facultatea de Chimie si Inginerie Chimica, Universitatea Babes-Bolyai, RO-400028, Cluj-Napoca, Romania; b Institut für Anorganische und Physikalische Chemie, Universität Bremen, D-28334, Bremen, Germany

## Abstract

The mol­ecular structure of the title compound, [Bi_2_(C_11_H_16_N)_4_(CO_3_)], consists of a symmetrically bridging carbonato group which binds two [2-Et_2_NCH_2_C_6_H_4_]_2_Bi units that are crystallographically related *via* a twofold rotation axis bis­ecting the carbonate group. The two Bi atoms and two of the C atoms directly bonded to bis­muth are quasi-planar [deviations of 0.323 (1) and 0.330 (9)Å for the Bi and C atoms, respectively] with the carbonate group. The remaining two ligands are in a *trans* arrangement relative to the quasi-planar (CBi)_2_CO_3_ system. The metal atom is strongly coordinated by the N atom of one pendant arm [Bi—N = 2.739 (6) Å], almost *trans* to the O atom, while the N atom of the other pendant arm exhibits a weaker intra­molecular inter­action [Bi⋯N = 3.659 (7) Å] almost *trans* to a C atom. If both these intra­molecular N→Bi inter­actions per metal atom are considered, the overall coordination geometry at bis­muth becomes distorted square-pyramidal [(C,N)_2_BiO cores] and the compound can be described as a hypervalent 12-*Bi*-5 species. Additional quite short intra­molecular Bi⋯O inter­actions are also present [3.796 (8)–4.020 (9) Å]. Inter­molecular associations through weak η^6^⋯Bi inter­actions [Bi⋯centroid of benzene ring = 3.659 (1) Å] lead to a ribbon-like supra­molecular association.

## Related literature

For structures of related carbonates and similar η^6^⋯Bi inter­actions, see: Breunig *et al.* (2008 ▶, 2010 ▶); Yin *et al.* (2008 ▶). For the chirality induced by the coordination of the N atom, see: IUPAC (1979 ▶). For Bi—N, Bi—O and Bi—C bond lengths, see: Emsley, (1994 ▶). For CO_2_ absorption by bis(diorganobismuth)oxides, see: Suzuki *et al.* (1994 ▶).
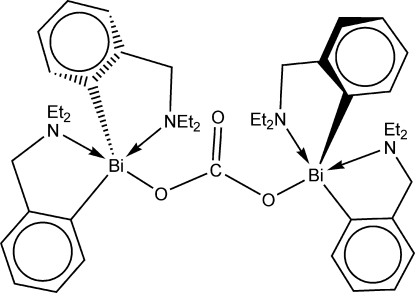


## Experimental

### 

#### Crystal data

[Bi_2_(C_11_H_16_N)_4_(CO_3_)]
*M* *_r_* = 1126.96
Monoclinic, 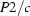

*a* = 12.263 (3) Å
*b* = 12.785 (2) Å
*c* = 15.286 (2) Å
β = 105.94 (1)°
*V* = 2304.4 (7) Å^3^
*Z* = 2
Mo *K*α radiation
μ = 7.67 mm^−1^
*T* = 173 K
0.60 × 0.30 × 0.20 mm

#### Data collection

Siemens P4 diffractometer
Absorption correction: refined from Δ*F* (Walker & Stuart, 1983 ▶) *T*_min_ = 0.076, *T*_max_ = 0.526
9290 measured reflections
4065 independent reflections
3351 reflections with *I* > 2σ(*I*)
*R*_int_ = 0.047
3 standard reflections every 197 reflections  intensity decay: 2%

#### Refinement

*R*[*F* ^2^ > 2σ(*F* ^2^)] = 0.041
*wR*(*F* ^2^) = 0.104
*S* = 1.01
4065 reflections
243 parameters
H-atom parameters constrained
Δρ_max_ = 1.83 e Å^−3^
Δρ_min_ = −2.12 e Å^−3^

### 

Data collection: *XSCANS* (Siemens, 1994 ▶); cell refinement: *XSCANS*; data reduction: *XSCANS*; program(s) used to solve structure: *SHELXS97* (Sheldrick, 2008 ▶); program(s) used to refine structure: *SHELXL97* (Sheldrick, 2008 ▶); molecular graphics: *DIAMOND* (Brandenburg, 2006 ▶); software used to prepare material for publication: *enCIFer* (Allen *et al.*, 2004 ▶) and *publCIF* (Westrip, 2010 ▶).

## Supplementary Material

Crystal structure: contains datablocks I, global. DOI: 10.1107/S160053681005453X/rk2252sup1.cif

Structure factors: contains datablocks I. DOI: 10.1107/S160053681005453X/rk2252Isup2.hkl

Additional supplementary materials:  crystallographic information; 3D view; checkCIF report

## Figures and Tables

**Fig. 1.**
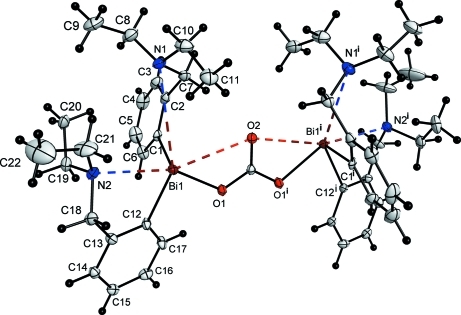
ORTEP representation of title complex (SN1,RN2/SN1i,RN2i-isomer) with the atom numbering scheme. Displacement ellipsoids are presented at 30% probability level. H atoms are shown as small spheres of arbitrary radius. Intramolecular N···Bi and O···Bi interactions are drawn by dashed lines. Symmetry code: (i) 2-x, y, 1/2-z.

**Fig. 2.**
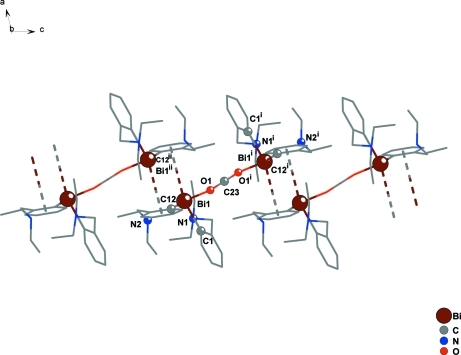
View along the b axis showing the η6···Bi interactions between alternating (RN1,SN2/RN1i,SN2i) and (SN1,RN2/SN1i,RN2i) isomers. Hydrogen atoms are omitted for clarity. Symmetry codes: (i) 2-x, y, 1/2-z; (ii) 2-x, 1-y, -z.

## supplementary crystallographic information

### Comment

The title compound was obtained by reaction of Na_2_CO_3_ with the corresponding diorganobismuth(III) chloride, [2-(Et_2_NCH_2_)C_6_H_4_]_2_BiCl in 1:2 molar ratio, in CH_2_Cl_2_/H_2_O mixture. The compound was also obtained when *R*_2_BiCl [*R* = 2-(Et_2_NCH_2_)C_6_H_4_] is reacted with KOH in toluene/water in open atmosphere, following the slow absorption of CO_2_ from atmosphere by the bis(diorganobismuth)oxide, as reported for *Ph*_3_BiCO_3_ (Suzuki *et al.*, 1994) or related hypervalent compound (Breunig *et al.*, 2008; Yin *et al.*, 2008).

The room temperature ^1^H NMR-spectra in CDCl_3_-d_1_ or toluene-d_8_ of the title compound exhibits only one set of resonances for the two organic groups attached to the bismuth centre, thus suggesting their equivalence at the NMR time scale. The ^1^H-resonances observed for the ethyl group protons and the AB spin systems for the benzylic CH_2_ protons are consistent with the coordination of both nitrogen atoms to the metal centre in solution. The coalescence of the AB system of the benzylic protons is not reached even at 333 K, thus indicating configurational stability.

The molecular structure of the title compound consists of a symmetrically bridging carbonate group [Bi1–O1/Bi1^i^–O1^i^ 2.239 (4)Å; Σ*r*_cov_(Bi,*O*) 2.18Å] which binds two [2-Et_2_NCH_2_C_6_H_4_]_2_Bi moieties that are crystallographically related *via* a twofold rotation axis bisecting the planar carbonate group. The two Bi atoms and two of the C atoms directly bonded to Bi are quasi-planar with the carbonate group [deviations from the CO_3_ plane: Bi1/Bi1^i^±0.323 (1)Å and C12/C12^i^±0.330 (9)Å]. The remaining two ligands are in *trans*-arrangement relative to the quasi-planar (CBi)_2_CO_3_ system. The metal centre is strongly coordinated by the nitrogen atom of one pendant arm [Bi1···N2 = 2.739 (6)Å; *c.f.* sums of the corresponding covalent, Σ*r*_cov_(Bi,*N*) = 2.22Å, and van der Waals radii, Σ*r*_vdW_(Bi,*N*) = 3.94Å (Emsley, 1994)], almost *trans* to the oxygen atom [N2···Bi1–O1 152.6 (2)°], while the nitrogen atom of the other pendant arm exhibits a weaker intramolecular interaction [Bi1···N1 = 3.659 (7)Å] almost *trans* to a carbon atom [angle N1···Bi1–C12 = 145.5 (2)°]. If both these intramolecular N→Bi interactions per metal atom are considered, the overall coordination becomes distorted square-pyramidal [(C,N)_2_BiO cores], with C1 in apical position and the compound can be described as hypervalent 12-Bi-5 species. Additional quite short intramolecular Bi···O interactions are also present [Bi1···O2 = 3.030 (2)Å; *c.f.*Σ*r*_vdW_(Bi,*O*) = 3.8Å] (Fig.1). Similar or structurally related carbonate coordination patterns have only recently been reported for hypervalent organobismuth(III) compounds (Breunig *et al.*, 2008, 2010; Yin *et al.*, 2008).

Coordination of N atom induces planar chirality, with the phenyl ring as chiral plane and the nitrogen as pilot atom (IUPAC, 1979). The compound crystalizes as a racemate.

Intermolecular associations through weak [η^6^···Bi interactions [Bi1···*Cg*2 = 3.659 (1)Å (*Cg*2 centroid of ring defined by C12^ii^-C17^ii^ atoms; Bi···*Cg* distance range 3.796 (8)-4.020 (9)Å; *c.f.* sums of the corresponding van der Waals radii Σ*r*_vdW_(Bi,*C*_sp2_) = 4.25Å, (Emsley, 1994)] between alternating (*R*_N1_,*S*_N2_/*R*_N1^i^_,*S*_N2^i^_) and (*S*_N1_,*R*_N2_*/S*_N1^i^_*R*_N2^i^_) isomers lead to a ribbon-like supramolecular association (Fig. 2). The η^6^···Bi interactions are almost *trans* to a Bi–C bond [C1–Bi1···*Cg*2 = 165.2 (2)°]. Symmetry codes: (i) 2-*x*, *y*, 1/2-*z*; (ii) 2-*x*, 1-*y*, -*z*). Similar η^6^···Bi interactions have been reported for [{2-(*Me*_2_NCH_2_)C_6_H_4_}_2_Bi]_2_CO_3_^.^C_6_H_6 _(Breunig *et al.*, 2008).

### Experimental

To a stirred solution of [2-(Et_2_NCH_2_)C_6_H_4_]_2_BiCl (1 g, 1.75 mmoles) in 35 ml CH_2_Cl_2_ was added, at room temperature, a solution of Na_2_CO_3_ (0.09 g, 0.84 mmoles + 0.09 g, 100% excess) in 25 ml distilled water. The reaction mixture was stirred for 48 h at room temperature, then the organic layer was separated and the water phase was washed with CH_2_Cl_2_ (2 × 40 ml). The resulting colourless solution was dried on Na_2_SO_4_ for 24 h and then filtered. Evaporation of the solution under vacuum afforded the title compound: 0.62 g (65%) as a white powder. Single crystals were grown from CH_2_Cl_2_ / *n*-hexane (1:5 by volume). *M.p.* = 393 K. Elemental analyses: found: C, 48.23; H, 5.65; N, 4.87. Calc. for C_45_H_64_Bi_2_N_4_O_3_: C, 47.96; H, 5.72; N 4.97%. IR (nujol, cm^-1^): ν(CO_3_) 1656 s, 1877 s. ^1^H-NMR (CDCl_3_, 300.11 MHz, r.t.): δ 0.92 t (24*H*, -NCH_2_C*H*_3_, ^3^*J*_HH_ = 7.1 Hz), 2.57 q (8*H*, -NC*H*_2_CH_3_, ^3^*J*_HH_ = 7.1 Hz), AB spin system with A at 3.60 and B at 3.80 (8*H*, -C*H*_2_-, ^2^*J*_HH_ = 13.3 Hz), 7.25 m (8*H*, *H*_4_ + *H*_5_), 7.40 m (4*H*, *H*_3_), 8.24 d (4*H*, *H*_6_, ^3^*J*_HH_ = 7.2 Hz). ^13^C-NMR (CDCl_3_, 75.47 MHz, r.t.): δ 9.72 s (-NCH_2_*C*H_3_), 44.95 s (-N*C*H_2_CH_3_), 61.61 s (*C*_7_), 127.15 s (*C*_4_), 129.51 s (*C*_3_), 130.06 s (*C*_5_), 139.35 s (*C*_6_), 146.49 s (*C*_2_), 164.99 s [O–*C*(O)–O], 188.32 s (*C*_1_). MS (CI*_pos_*, NH_3_, 473 K): *m/z*, (relative intensity, %) 776 (1) [(*R*BiO)_2_]^+^, 533 (100) [*R*_2_Bi]^+^, 386 (4) [*R*BiO]^+^, 164 [*R* + 2H]^+^. MS (CI*_neg_*, NH_3_, 473 K): *m/z*, (relative intensity, %) 549 (10) [*R*_2_BiO]^-^, 533 (18) [*R*_2_Bi]^-^.

### Refinement

All hydrogen atoms were placed in calculated positions using a riding model, with C–H = 0.93-0.97Å and with *U*_iso_ = 1.5*U*_eq_ (C) for methyl H and *U*_iso_ = 1.2*U*_eq_(C) for non-mehyl H. Residual electron densities are 1.68 e.Å^-3^ at 0.1171 0.6442 0.0513 (1.53Å from N2) and -2.09 e.Å^-3^ at 0.1656 0.6430 0.0413 (1.35Å from N2). In the crystal structure there is a 37Å^3^ void (0.500 0.028 0.750 - 3.17Å from C9), but the low electron density (0.19 e.Å^-3^) in the difference Fourier map suggests no solvent molecule occupying this void.

### Crystal data

**Table:**

[Bi_2_(C_11_H_16_N)_4_(CO_3_)]	*F*(000) = 1104
*M**_r_* = 1126.96	*D*_x_ = 1.624 Mg m^−^^3^
Monoclinic, *P*2/*c*	Melting point: 393 K
Hall symbol: -P 2yc	Mo *K*α radiation, λ = 0.71073 Å
*a* = 12.263 (3) Å	Cell parameters from 37 reflections
*b* = 12.785 (2) Å	θ = 4.9–25.0°
*c* = 15.286 (2) Å	µ = 7.67 mm^−^^1^
β = 105.94 (1)°	*T* = 173 K
*V* = 2304.4 (7) Å^3^	Block, colourless
*Z* = 2	0.60 × 0.30 × 0.20 mm

### Data collection

**Table:**

Siemens P4 diffractometer	3351 reflections with *I* > 2σ(*I*)
Radiation source: sealed tube	*R*_int_ = 0.047
graphite	θ_max_ = 25.0°, θ_min_ = 2.5°
2θ/ω scans	*h* = −14→7
Absorption correction: part of the refinement model (Δ*F*) (Walker & Stuart, 1983)	*k* = −15→15
*T*_min_ = 0.076, *T*_max_ = 0.526	*l* = −17→18
9290 measured reflections	3 standard reflections every 197 reflections
4065 independent reflections	intensity decay: 2%

### Refinement

**Table:**

Refinement on *F*^2^	Primary atom site location: structure-invariant direct methods
Least-squares matrix: full	Secondary atom site location: difference Fourier map
*R*[*F*^2^ > 2σ(*F*^2^)] = 0.041	Hydrogen site location: inferred from neighbouring sites
*wR*(*F*^2^) = 0.104	H-atom parameters constrained
*S* = 1.01	*w* = 1/[σ^2^(*F*_o_^2^) + (0.0659*P*)^2^] where *P* = (*F*_o_^2^ + 2*F*_c_^2^)/3
4065 reflections	(Δ/σ)_max_ = 0.001
243 parameters	Δρ_max_ = 1.83 e Å^−^^3^
0 restraints	Δρ_min_ = −2.12 e Å^−^^3^

### Special details

**Table:**

Geometry. All s.u.'s (except the s.u. in the dihedral angle between two l.s. planes) are estimated using the full covariance matrix. The cell s.u.'s are taken into account individually in the estimation of s.u.'s in distances, angles and torsion angles; correlations between s.u.'s in cell parameters are only used when they are defined by crystal symmetry. An approximate (isotropic) treatment of cell s.u.'s is used for estimating s.u.'s involving l.s. planes.
Refinement. Refinement of *F*^2^ against ALL reflections. The weighted *R*-factor *wR* and goodness of fit *S* are based on *F*^2^, conventional *R*-factors *R* are based on *F*, with *F* set to zero for negative *F*^2^. The threshold expression of *F*^2^ > σ(*F*^2^) is used only for calculating *R*-factors(gt) *etc*. and is not relevant to the choice of reflections for refinement. *R*-factors based on *F*^2^ are statistically about twice as large as those based on *F*, and *R*-factors based on ALL data will be even larger.

### Fractional atomic coordinates and isotropic or equivalent isotropic displacement parameters (Å^2^)

**Table:**

	*x*	*y*	*z*	*U*_iso_*/*U*_eq_	
Bi1	0.88618 (2)	0.358681 (18)	0.053526 (14)	0.01998 (12)	
C1	0.7204 (6)	0.3360 (5)	0.0910 (4)	0.0204 (14)	
C2	0.6990 (7)	0.2406 (6)	0.1311 (4)	0.0282 (17)	
C3	0.5970 (8)	0.2327 (7)	0.1543 (5)	0.039 (2)	
H3	0.5803	0.1709	0.1800	0.047*	
C4	0.5218 (8)	0.3126 (9)	0.1405 (6)	0.051 (3)	
H4	0.4550	0.3051	0.1576	0.062*	
C5	0.5425 (9)	0.4052 (8)	0.1013 (6)	0.050 (2)	
H5	0.4898	0.4594	0.0911	0.060*	
C6	0.6429 (7)	0.4161 (7)	0.0775 (5)	0.0332 (18)	
H6	0.6582	0.4786	0.0521	0.040*	
C7	0.7840 (8)	0.1526 (5)	0.1513 (5)	0.0313 (18)	
H7A	0.8586	0.1815	0.1792	0.038*	
H7B	0.7655	0.1059	0.1952	0.038*	
C8	0.6832 (10)	0.0306 (7)	0.0375 (6)	0.051 (3)	
H8A	0.6195	0.0766	0.0348	0.062*	
H8B	0.6821	−0.0241	0.0812	0.062*	
C9	0.6676 (11)	−0.0192 (8)	−0.0557 (6)	0.066 (3)	
H9A	0.6701	0.0340	−0.0994	0.099*	
H9B	0.5957	−0.0543	−0.0738	0.099*	
H9C	0.7273	−0.0689	−0.0527	0.099*	
C10	0.8872 (10)	0.0226 (7)	0.0953 (6)	0.051 (3)	
H10A	0.8787	−0.0314	0.0494	0.061*	
H10B	0.8892	−0.0114	0.1524	0.061*	
C11	0.9977 (9)	0.0769 (7)	0.1053 (6)	0.049 (2)	
H11A	1.0003	0.1041	0.0474	0.074*	
H11B	1.0587	0.0281	0.1270	0.074*	
H11C	1.0052	0.1333	0.1480	0.074*	
C12	0.8403 (7)	0.5173 (5)	−0.0111 (4)	0.0248 (16)	
C13	0.8200 (7)	0.5211 (5)	−0.1059 (4)	0.0248 (16)	
C14	0.7937 (7)	0.6172 (6)	−0.1503 (5)	0.0277 (16)	
H14	0.7774	0.6202	−0.2134	0.033*	
C15	0.7918 (7)	0.7076 (6)	−0.1014 (4)	0.0280 (16)	
H15	0.7761	0.7715	−0.1314	0.034*	
C16	0.8130 (7)	0.7034 (6)	−0.0077 (5)	0.0316 (18)	
H16	0.8110	0.7642	0.0251	0.038*	
C17	0.8372 (7)	0.6090 (6)	0.0367 (5)	0.0284 (17)	
H17	0.8516	0.6067	0.0997	0.034*	
C18	0.8293 (8)	0.4243 (6)	−0.1593 (5)	0.0310 (17)	
H18A	0.7928	0.4363	−0.2233	0.037*	
H18B	0.9086	0.4088	−0.1526	0.037*	
C19	0.6523 (8)	0.3465 (6)	−0.1538 (5)	0.037 (2)	
H19A	0.6271	0.3559	−0.2192	0.044*	
H19B	0.6338	0.4098	−0.1260	0.044*	
C20	0.5867 (8)	0.2568 (7)	−0.1285 (5)	0.044 (2)	
H20A	0.5886	0.1980	−0.1671	0.066*	
H20B	0.5095	0.2778	−0.1362	0.066*	
H20C	0.6204	0.2377	−0.0662	0.066*	
C21	0.8230 (14)	0.2316 (8)	−0.1560 (7)	0.087 (3)	
H21A	0.9051	0.2348	−0.1353	0.105*	
H21B	0.7991	0.1733	−0.1251	0.105*	
C22	0.7903 (14)	0.2099 (9)	−0.2524 (7)	0.087 (3)	
H22A	0.7095	0.2023	−0.2735	0.131*	
H22B	0.8260	0.1464	−0.2637	0.131*	
H22C	0.8138	0.2666	−0.2842	0.131*	
C23	1.0000	0.4009 (8)	0.2500	0.022 (2)	
N1	0.7887 (6)	0.0909 (5)	0.0702 (4)	0.0340 (15)	
N2	0.7747 (6)	0.3336 (5)	−0.1268 (4)	0.0302 (15)	
O1	0.9491 (5)	0.4556 (4)	0.1793 (3)	0.0277 (12)	
O2	1.0000	0.3026 (5)	0.2500	0.0249 (15)	

### Atomic displacement parameters (Å^2^)

**Table:**

	*U*^11^	*U*^22^	*U*^33^	*U*^12^	*U*^13^	*U*^23^
Bi1	0.02017 (17)	0.01928 (16)	0.02040 (16)	0.00227 (12)	0.00542 (11)	−0.00038 (9)
C1	0.008 (3)	0.030 (4)	0.023 (3)	0.000 (3)	0.003 (3)	0.000 (3)
C2	0.027 (4)	0.037 (4)	0.021 (3)	−0.007 (4)	0.008 (3)	−0.006 (3)
C3	0.036 (5)	0.049 (5)	0.036 (4)	−0.011 (4)	0.015 (4)	−0.004 (4)
C4	0.022 (5)	0.090 (8)	0.047 (5)	−0.001 (5)	0.017 (4)	−0.003 (5)
C5	0.039 (6)	0.068 (7)	0.044 (5)	0.018 (5)	0.015 (4)	−0.002 (5)
C6	0.031 (5)	0.042 (4)	0.029 (4)	0.009 (4)	0.011 (3)	0.002 (3)
C7	0.033 (5)	0.031 (4)	0.031 (4)	−0.008 (4)	0.009 (3)	0.001 (3)
C8	0.060 (7)	0.041 (5)	0.052 (5)	−0.016 (5)	0.014 (5)	−0.013 (4)
C9	0.081 (9)	0.037 (5)	0.078 (7)	−0.019 (6)	0.017 (7)	−0.020 (5)
C10	0.066 (8)	0.032 (5)	0.057 (5)	0.001 (5)	0.022 (5)	0.000 (4)
C11	0.053 (7)	0.039 (5)	0.056 (5)	0.009 (5)	0.015 (5)	−0.003 (4)
C12	0.028 (4)	0.022 (4)	0.020 (3)	0.006 (3)	−0.001 (3)	0.000 (3)
C13	0.031 (5)	0.022 (4)	0.023 (3)	0.003 (3)	0.009 (3)	0.000 (3)
C14	0.030 (4)	0.030 (4)	0.024 (3)	0.000 (4)	0.009 (3)	0.002 (3)
C15	0.021 (4)	0.027 (4)	0.036 (4)	0.003 (3)	0.008 (3)	0.008 (3)
C16	0.032 (5)	0.022 (4)	0.044 (4)	0.003 (4)	0.016 (4)	−0.005 (3)
C17	0.030 (5)	0.027 (4)	0.026 (3)	0.010 (4)	0.003 (3)	0.000 (3)
C18	0.042 (5)	0.028 (4)	0.027 (3)	−0.006 (4)	0.016 (3)	−0.007 (3)
C19	0.035 (5)	0.043 (5)	0.026 (4)	−0.003 (4)	−0.003 (4)	−0.003 (3)
C20	0.034 (5)	0.058 (6)	0.033 (4)	−0.018 (5)	−0.002 (4)	0.001 (4)
C21	0.154 (11)	0.043 (4)	0.078 (5)	0.002 (6)	0.051 (6)	−0.019 (4)
C22	0.154 (11)	0.043 (4)	0.078 (5)	0.002 (6)	0.051 (6)	−0.019 (4)
C23	0.016 (5)	0.025 (5)	0.023 (5)	0.000	0.003 (4)	0.000
N1	0.038 (4)	0.025 (3)	0.038 (3)	−0.009 (3)	0.010 (3)	−0.004 (3)
N2	0.030 (4)	0.031 (3)	0.029 (3)	−0.007 (3)	0.005 (3)	−0.008 (3)
O1	0.036 (3)	0.025 (3)	0.018 (2)	−0.001 (2)	−0.001 (2)	−0.0007 (18)
O2	0.023 (4)	0.026 (4)	0.024 (3)	0.000	0.003 (3)	0.000

### Geometric parameters (Å, °)

**Table:**

Bi1—O1	2.238 (4)	C12—C17	1.387 (10)
Bi1—C12	2.259 (7)	C12—C13	1.402 (9)
Bi1—C1	2.277 (7)	C13—C14	1.398 (9)
Bi1—N2	2.739 (6)	C13—C18	1.504 (9)
C1—C6	1.373 (10)	C14—C15	1.381 (10)
C1—C2	1.422 (10)	C14—H14	0.9300
C2—C3	1.393 (11)	C15—C16	1.384 (9)
C2—C7	1.508 (11)	C15—H15	0.9300
C3—C4	1.354 (13)	C16—C17	1.377 (10)
C3—H3	0.9300	C16—H16	0.9300
C4—C5	1.382 (15)	C17—H17	0.9300
C4—H4	0.9300	C18—N2	1.491 (10)
C5—C6	1.383 (13)	C18—H18A	0.9700
C5—H5	0.9300	C18—H18B	0.9700
C6—H6	0.9300	C19—N2	1.453 (11)
C7—N1	1.484 (9)	C19—C20	1.510 (11)
C7—H7A	0.9700	C19—H19A	0.9700
C7—H7B	0.9700	C19—H19B	0.9700
C8—N1	1.470 (12)	C20—H20A	0.9600
C8—C9	1.525 (11)	C20—H20B	0.9600
C8—H8A	0.9700	C20—H20C	0.9600
C8—H8B	0.9700	C21—C22	1.444 (13)
C9—H9A	0.9600	C21—N2	1.548 (12)
C9—H9B	0.9600	C21—H21A	0.9700
C9—H9C	0.9600	C21—H21B	0.9700
C10—N1	1.454 (12)	C22—H22A	0.9600
C10—C11	1.492 (14)	C22—H22B	0.9600
C10—H10A	0.9700	C22—H22C	0.9600
C10—H10B	0.9700	C23—O2	1.257 (13)
C11—H11A	0.9600	C23—O1^i^	1.295 (7)
C11—H11B	0.9600	C23—O1	1.295 (7)
C11—H11C	0.9600		
			
O1—Bi1—C12	82.2 (2)	C14—C13—C18	120.1 (6)
O1—Bi1—C1	88.6 (2)	C12—C13—C18	121.0 (6)
C12—Bi1—C1	95.3 (3)	C15—C14—C13	120.6 (6)
O1—Bi1—N2	152.65 (18)	C15—C14—H14	119.7
C12—Bi1—N2	70.7 (2)	C13—C14—H14	119.7
C1—Bi1—N2	90.3 (2)	C14—C15—C16	120.1 (6)
C6—C1—C2	120.1 (7)	C14—C15—H15	120.0
C6—C1—Bi1	119.7 (5)	C16—C15—H15	120.0
C2—C1—Bi1	120.1 (5)	C17—C16—C15	119.9 (7)
C3—C2—C1	117.2 (8)	C17—C16—H16	120.0
C3—C2—C7	120.8 (7)	C15—C16—H16	120.0
C1—C2—C7	122.0 (7)	C16—C17—C12	120.9 (6)
C4—C3—C2	121.8 (8)	C16—C17—H17	119.5
C4—C3—H3	119.1	C12—C17—H17	119.5
C2—C3—H3	119.1	N2—C18—C13	110.6 (6)
C3—C4—C5	121.0 (9)	N2—C18—H18A	109.5
C3—C4—H4	119.5	C13—C18—H18A	109.5
C5—C4—H4	119.5	N2—C18—H18B	109.5
C4—C5—C6	118.9 (9)	C13—C18—H18B	109.5
C4—C5—H5	120.6	H18A—C18—H18B	108.1
C6—C5—H5	120.6	N2—C19—C20	115.0 (7)
C1—C6—C5	121.0 (8)	N2—C19—H19A	108.5
C1—C6—H6	119.5	C20—C19—H19A	108.5
C5—C6—H6	119.5	N2—C19—H19B	108.5
N1—C7—C2	114.2 (6)	C20—C19—H19B	108.5
N1—C7—H7A	108.7	H19A—C19—H19B	107.5
C2—C7—H7A	108.7	C19—C20—H20A	109.5
N1—C7—H7B	108.7	C19—C20—H20B	109.5
C2—C7—H7B	108.7	H20A—C20—H20B	109.5
H7A—C7—H7B	107.6	C19—C20—H20C	109.5
N1—C8—C9	114.2 (8)	H20A—C20—H20C	109.5
N1—C8—H8A	108.7	H20B—C20—H20C	109.5
C9—C8—H8A	108.7	C22—C21—N2	115.8 (10)
N1—C8—H8B	108.7	C22—C21—H21A	108.3
C9—C8—H8B	108.7	N2—C21—H21A	108.3
H8A—C8—H8B	107.6	C22—C21—H21B	108.3
C8—C9—H9A	109.5	N2—C21—H21B	108.3
C8—C9—H9B	109.5	H21A—C21—H21B	107.4
H9A—C9—H9B	109.5	C21—C22—H22A	109.5
C8—C9—H9C	109.5	C21—C22—H22B	109.5
H9A—C9—H9C	109.5	H22A—C22—H22B	109.5
H9B—C9—H9C	109.5	C21—C22—H22C	109.5
N1—C10—C11	114.4 (7)	H22A—C22—H22C	109.5
N1—C10—H10A	108.7	H22B—C22—H22C	109.5
C11—C10—H10A	108.7	O2—C23—O1^i^	122.7 (4)
N1—C10—H10B	108.7	O2—C23—O1	122.7 (4)
C11—C10—H10B	108.7	O1^i^—C23—O1	114.7 (9)
H10A—C10—H10B	107.6	C10—N1—C8	111.4 (7)
C10—C11—H11A	109.5	C10—N1—C7	108.6 (6)
C10—C11—H11B	109.5	C8—N1—C7	109.3 (7)
H11A—C11—H11B	109.5	C19—N2—C18	109.9 (6)
C10—C11—H11C	109.5	C19—N2—C21	117.5 (8)
H11A—C11—H11C	109.5	C18—N2—C21	108.5 (7)
H11B—C11—H11C	109.5	C19—N2—Bi1	117.6 (5)
C17—C12—C13	119.5 (6)	C18—N2—Bi1	95.6 (4)
C17—C12—Bi1	124.6 (5)	C21—N2—Bi1	105.3 (6)
C13—C12—Bi1	115.8 (5)	C23—O1—Bi1	113.1 (5)
C14—C13—C12	118.9 (6)		
			
O1—Bi1—C1—C6	75.9 (5)	C13—C12—C17—C16	0.9 (13)
C12—Bi1—C1—C6	−6.1 (6)	Bi1—C12—C17—C16	177.2 (6)
N2—Bi1—C1—C6	−76.7 (5)	C14—C13—C18—N2	−138.3 (8)
O1—Bi1—C1—C2	−101.8 (5)	C12—C13—C18—N2	43.4 (11)
C12—Bi1—C1—C2	176.1 (5)	C11—C10—N1—C8	164.4 (7)
N2—Bi1—C1—C2	105.5 (5)	C11—C10—N1—C7	−75.1 (9)
C6—C1—C2—C3	0.8 (10)	C9—C8—N1—C10	−71.3 (10)
Bi1—C1—C2—C3	178.6 (5)	C9—C8—N1—C7	168.7 (8)
C6—C1—C2—C7	−177.0 (6)	C2—C7—N1—C10	169.2 (7)
Bi1—C1—C2—C7	0.8 (9)	C2—C7—N1—C8	−69.0 (8)
C1—C2—C3—C4	−0.8 (12)	C20—C19—N2—C18	177.3 (6)
C7—C2—C3—C4	177.0 (7)	C20—C19—N2—C21	52.6 (9)
C2—C3—C4—C5	1.0 (14)	C20—C19—N2—Bi1	−74.8 (7)
C3—C4—C5—C6	−1.1 (14)	C13—C18—N2—C19	71.0 (8)
C2—C1—C6—C5	−1.0 (11)	C13—C18—N2—C21	−159.3 (8)
Bi1—C1—C6—C5	−178.8 (6)	C13—C18—N2—Bi1	−51.0 (7)
C4—C5—C6—C1	1.1 (13)	C22—C21—N2—C19	56.3 (14)
C3—C2—C7—N1	105.3 (8)	C22—C21—N2—C18	−69.1 (13)
C1—C2—C7—N1	−76.9 (9)	C22—C21—N2—Bi1	−170.6 (10)
O1—Bi1—C12—C17	−14.3 (7)	O1—Bi1—N2—C19	−69.1 (7)
C1—Bi1—C12—C17	73.5 (7)	C12—Bi1—N2—C19	−77.1 (6)
N2—Bi1—C12—C17	162.0 (8)	C1—Bi1—N2—C19	18.4 (6)
O1—Bi1—C12—C13	162.1 (6)	O1—Bi1—N2—C18	46.9 (7)
C1—Bi1—C12—C13	−110.0 (6)	C12—Bi1—N2—C18	38.9 (5)
N2—Bi1—C12—C13	−21.6 (5)	C1—Bi1—N2—C18	134.4 (5)
C17—C12—C13—C14	−1.9 (12)	O1—Bi1—N2—C21	157.8 (6)
Bi1—C12—C13—C14	−178.5 (6)	C12—Bi1—N2—C21	149.8 (7)
C17—C12—C13—C18	176.4 (8)	C1—Bi1—N2—C21	−114.7 (6)
Bi1—C12—C13—C18	−0.2 (10)	O2—C23—O1—Bi1	−9.0 (4)
C12—C13—C14—C15	2.4 (13)	O1^i^—C23—O1—Bi1	171.0 (4)
C18—C13—C14—C15	−176.0 (8)	C12—Bi1—O1—C23	−175.1 (4)
C13—C14—C15—C16	−1.7 (13)	C1—Bi1—O1—C23	89.3 (4)
C14—C15—C16—C17	0.6 (12)	N2—Bi1—O1—C23	177.3 (3)
C15—C16—C17—C12	−0.2 (13)		

Symmetry codes: (i) −*x*+2, *y*, −*z*+1/2.
